# *In silico *discovery of human natural antisense transcripts

**DOI:** 10.1186/1471-2105-7-18

**Published:** 2006-01-13

**Authors:** Yuan-Yuan Li, Lei Qin, Zong-Ming Guo, Lei Liu, Hao Xu, Pei Hao, Jiong Su, Yixiang Shi, Wei-Zhong He, Yi-Xue Li

**Affiliations:** 1 Shanghai Center for Bioinformation Technology, Shanghai 200235, China; 2 The W. M. Keck Center for Comparative and Functional Genomics, University of Illinois at Urbana-Champaign, 1201 W. Gregory Dr., Urbana, Illinois 61801, USA

## Abstract

**Background:**

Several high-throughput searches for ppotential natural antisense transcripts (NATs) have been performed recently, but most of the reports were focused on *cis *type. A thorough *in silico *analysis of human transcripts will help expand our knowledge of NATs.

**Results:**

We have identified 568 NATs from human RefSeq RNA sequences. Among them, 403 NATs are reported for the first time, and at least 157 novel NATs are *trans *type. According to the pairing region of a sense and antisense RNA pair, hNATs are divided into 6 classes, of which about 87% involve 5' or 3' UTR sequences, supporting the regulatory role of UTRs. Among a total of 535 NAT pairs related with splice variants, 77.4% (414/535) have their pairing regions affected or completely eliminated by alternative splicing, suggesting significant relationship of alternative splicing and antisense-directed regulation. The extensive occurrence of splice variants in hNATs and other multiple pairing patterns results in a one-to-many relationship, allowing the formation of complex regulation networks. Based on microarray data from Stanford Microarray Database, two hNAT pairs were found to display significant inverse expression patterns before and after insulin injection.

**Conclusion:**

NATs might carry out more extensive and complex functions than previously thought. Combined with endogenous micro RNAs, hNATs could be regarded as a special group of transcripts contributing to the complex regulation networks.

## Background

Natural antisense transcripts (NATs) are endogenous ones that exhibit complementary sequences to transcripts of a known function, or sense transcripts. NATs were first described in prokaryotes [1], and they were found to down-regulate the expression of sense transcripts involved in diverse biological functions, such as transposition, plasmid replication and gene expression [2]. Since the discovery of first NAT in human [3], an increasing number of NATs in mammalian organisms have been reported to be related to genomic imprinting [4], RNA interference [5], alternative splicing [6], X-inactivation [7] and RNA editing [8]. It has been shown that NATs could perform two non-exclusive major functions: template for translation and regulation of sense gene expression, and the latter may occur at the level of transcription, maturation, transport, stability and/or translation [9].

As NATs seemed to exist so extensively, independent genome-wide searches for potential NATs were performed recently with data from RefSeq, UniGene or some specially constructed EST database. Due to differences in data sources and/or criteria used in searches, the number of reported human NATs (hNATs) varies greatly, from hundreds [10,11] to thousands [12,13].

There are two different types of NATs, *cis *and *trans*. A *cis *NAT is transcribed from the opposite strand of the same genomic locus as its sense RNA, thus the pair displays perfect sequence complementarity. In contrast, a *trans *NAT is transcribed from a genomic locus different from its sense counterpart and the pair may display imperfect complementarity [9]. All early works in human, mouse, and drosophila revealed only *cis *NATs [14-16]. Although increasing evidence suggests that *trans *NATs might perform more significant and versatile functions than previously expected [11], most of the high-throughput searches were focused on *cis *NATs but overlooked *trans *ones [10,12,13]. The bias towards *cis *type also existed in Kiyosawa *et al*'s survey on mouse genome [17]. Lehner *et al*. [11] investigated both *cis *and *trans *NATs based on available mRNAs, and found 372 human NATs including 80 putative *trans *ones. However, we believe that they underestimated the prevalence of hNATs since many known antisense RNAs are non-protein coding.

In this work, we carried out a thorough *in silico *analysis of human RNA sequences from the RefSeq database [18] and identified 568 hNATs, among which 403 NATs were reported for the first time. Out of the 313 novel NATs that have genomic mapping data available, we determined that 157 are *trans *NATs. We also classified the 568 NATs according to pairing regions and found that 87% cases involved UTR sequences of one or both transcripts. We noticed that among a total of 535 NAT pairs related with splice variants, 77.4% (414/535) have their pairing regions affected or completely eliminated by alternative splicing, suggesting significant relationship of alternative splicing and antisense-directed regulation. We proposed that the one-to-many relationship of sense and antisense transcript pairs, caused by the extensive occurrence of splice variants in hNATs and other multiple pairing patterns, may lead to a probable regulatory network. Furthermore, using Stanford Microarray Database (SMD) [19], we found two hNAT pairs displaying a significant inverse expression pattern before and after insulin injection.

## Results and Discussion

### Identification of hNATs from RefSeq with BLASTN

Previous large-scale hNATs searches have significantly expanded our knowledge about the prevalence of hNAT [10-13]. Particularly, Chen *et al*. [13] predicted that about 20% of the human genes formed sense-antisense transcript pairs.

In this work, we studied hNATs with RefSeq [18], which is a manually curated database that collects comprehensive, non-redundant sets of sequences, and identified 568 hNATs including both *cis *and *trans *types (see Table 1 for summary and Additional Table 1 for detailed information of each pair). Although we found limited hNATs compared to previous reports, sequence accession number matching showed that 403 out of the 568 NAT pairs did not appear in any of the published data sets [10-13], indicating that antisense regulation may be even more widespread in the mammalian genome than appreciated previously.

The reason that previous studies did not find these entries might be due to different starting data and/or the criteria used. Most of these studies identified NATs based on genomic locus overlapping. As a result, they tend to find *cis *NATs, but overlooked *trans *ones [10,12,13]. For example, Yelin *et al*. [12] mainly collected sequences that span intron(s) from human expressed sequences (mRNAs and expressed-sequence tags (ESTs)), and predicted 8.4% of human transcription clusters formed *cis *NAT pairs. Similarly, Chen *et al*. [13] investigated sense-orientation-reliable ESTs from UniGene, and got the largest *cis *hNATs data set reported to date. Non-polyadenylated transcripts were not included in their study, which may partly explain why they did not find the novel *cis *pairs in this work. We did not exclude the possibility that some of the novel *cis *pairs in this paper were actually included in Chen *et al*.'s results [13] since our comparison was based on accession number only and there are cases that a sequence entry acquires a different primary accession number in following releases due to sequence split or merge. Lehner *et al*. [11] identified both *trans *and *cis *hNATs, but they only considered mRNA, and ignored non-coding RNA which occurs in many known functional NAT examples. Moreover, the newer version of RefSeq database that we used in this work contains nearly twice as many entries of human mRNA (25,827) as that in the previous version (12,897) used by Lehner *et al*. [11]. As a result, we were able to identify more novel hNATs, particularly *trans *type NATs. The 568 hNATs are summarized in Table 1. Out of the 568 entries, 473 have genomic mapping data available in the Genome database. Thus we determined that 312 NATs are *cis *type and 161 are *trans *type (see Additional Table 1 for detailed information). One hundred and fifty seven out of the 161 *trans *NATs were reported for the first time. The breadth and importance of *trans *NATs-directed gene regulation are coming into focus as more *trans *encoded NATs, particularly small nucleolar RNAs (snoRNAs) and micro RNAs (miRNAs), are discovered [20-22]. To date, underlying mechanisms of *trans *NATs are relatively less understood than *cis *ones. The identification of more novel *trans *NATs in this work may shed some light on this field.

It is noticeable that Lehner *et al*. concluded that 51 out of their 80 *trans *hNATs were likely to be chimeric mRNA containing sequences from two different chromosomal loci due to artifacts of cDNA library construction and chromosomal rearrangements [11]. However, after carefully checking the *trans *hNATs they reported, we found that 21 out of the 23 RefSeq *trans *hNAT pairs thought to be involving suspected chimeras can now be mapped to certain loci, thus are true *trans *NATs. Among these 21 NATs, the chromosomal location information of 4 entries was modified in the Genome database, suggesting that the presence of *trans *NATs did affect the genome assembly and gene localization as people worried about. It seems that the temporal unfinished human genome assembly was a major obstacle in their survey on *trans *hNATs.

Our data do not cover all of previously reported ones [10-13]. This is due to our choice of using the RefSeq database and the stringent criteria for BLASTN (e-value cutoff of 1e-9 and identity above 98%). RefSeq includes both mRNAs and non-mRNAs, but it has a bias towards mRNA because of the convenience of identifying mRNA. There are 25827 human mRNA and 148 human non-mRNA entries in the release of RefSeq we used. Thus, our choice of using RefSeq helped to find coding antisense RNAs, while underestimated the non-coding antisense RNAs. For example, the first reported hNAT over the *c-myc *locus, a non-coding antisense transcript, cannot be found in our result [3].

Eighteen out of the 568 (3.2%) hNAT pairs we found involved non-coding RNAs, which is significantly higher than the expected 1.1%, assuming no bias for mRNA or non-mRNA to form NAT pairs. The expected value is estimated as 1–99.43%*99.43%, where 99.43% is the percentage of mRNA entries in the starting dataset. This indicates that NATs tend to be non-coding RNAs, supporting Chen *et al*.'s observations [13], while disagreeing with Lehner *et al*.'s assumption [11]. As new non-coding RNAs are being discovered, we believe that the number of reported NAT pairs will rise further.

Even though coding transcripts have fewer propensities to form NAT pairs in contrast to non-coding ones, they have been recently reported more frequently than before [9,23,24]. Since coding NATs function as both template and regulator, they may lead to more complex gene regulation, as the THRA (c-erbAα) and NR1D1 (Rev-erbAα) NAT pair example demonstrates below.

### Classification of hNATs based on the pairing region

Assuming that the complementarity of sense and antisense RNA pair is the major basis of the regulation function of antisense RNAs [9], we analyzed the overlapping length and location of these 568 hNATs. It was found that overlapping lengths varied from 34 bp to 1810 bp (see Additional Table 1), similar to previously reported [10-12]. We classified the 550 hNAT pairs that have CDS location data into 6 types according to the pairing region: 1) 5'UTR vs. 5'UTR, 2) 5'UTR vs. CDS, 3) 5'UTR vs. 3'UTR, 4) CDS vs. CDS, 5) CDS vs. 3'UTR, 6) 3'UTR vs. 3'UTR (Table 2). More than 87% of the 550 NAT pairs involved 5' or 3' UTR, that is, types 1, 2, 3, 5 and 6, supporting the significance of UTR in antisense-mediated regulation [9,25,26].

According to our data, the subtype 6-*cis*, or 3'UTR vs. 3'UTR-*cis*, is the most common form of overlapping. While, analysis of adjacent gene sets in *S. cerevisiae *suggested that there might be evolutionary pressure to select against convergent genes [27], and the overlapping arrangement of convergent genes restricted the elongation of both transcripts, resulting in a severe reduction in mRNA accumulation, termed as transcriptional collision [28]. This collision effect could be a direct physical impediment to the transcription machinery or an indirect effect caused by supercoiling changes to the DNA template during transcription, while seemed unrelated with interference of antisense RNAs [28]. The precise biological implication of the convergent arrangement of genes in yeast and mammalian genomes remain to be elucidated. The 6-*cis *dataset including 116 hNAT pairs might provide basis for future in-depth investigation.

The subtype 1-*cis*, or 5'UTR vs. 5'UTR-*cis *NAT pairs, could be involved in bidirectional transcription driven by a divergent promoter, which seems to be a preferred structure in prokaryotes for gene regulation such as transcription coupling [29]. Transcription coupling is different from the traditional concept of antisense phenomena, and the choice between transcription coupling and antisense transcript-directed inhibition may depend on the overlapping length and involved transcription factor binding sites. Since bi-directional transcription in eukaryotes is not as common as in prokaryotes, the precise organization and its significance of the 20 members of type 1-*cis *are intriguing. An in-depth analysis is still in progress.

### Splice variants involved in hNATs

Since the wide existence of alternative splicing has been well established [30], and there is evidence for the involvement of NATs in alternative splicing [31,32], we also investigated the splice variants involved in hNATs. In the present 568 NATs pairs, 63 genes involved in 168 NAT pairs have splice variants (see Additional Table 2A). Forty-nine of the 63 genes involved in 121 pairs have pairing regions unaffected by alternative splicing; while the other 14 genes involved in 47 pairs have variable pairing regions due to alternative splicing. As an example for the latter case, the SPAG8 gene has two splice variants, NM_012436 and NM_172312, and they may pair with antisense transcript NPR2 with 308 bp overlap of 99% identity, and 110 bp overlap of 100% identity, respectively. It is conceivable that alternative splicing can also make the whole pairing region lost. In case of the IL18BP gene (see Additional Table 2A), it has four splice variants, but only three of them have antisense transcript NUMA1. To see how frequently this can happen, we checked the rest 400 hNAT pairs (568-168) using AceView [33] and found that additional 367 NAT pairs involved splicing variants (see Additional Table 2B). In these cases, only one of the transcript variants pairs with its countertranscript while the others lose the pairing region because of alternative splicing. Therefore these cases were not contained in Additional Table 2A. Among the 535 NAT pairs (168+367) related with splice variants, 22.6% (121/535) have splicing variants sharing the same pairing region, 77.4% (414/535) have their pairing regions affected or completely eliminated by alternative splicing. The remarkably high percentage of the latter suggested significant relationship of alternative splicing and antisense-directed regulation. Taking the THRA (c-erbAα) and NR1D1 (Rev-erbAα) pair (No. 472 in the Additional Table 1) as an example, the sense transcript c-erbAα encodes two structurally related proteins R-erbAα1 and R-erbAα2 by alternative splicing [23]. The antisense rev-erbAα transcript is complementary to the last exon of r-erbAα2 mRNA but not to the r-erbAα1 mRNA. It was indicated that rev-erbAα messenger prevents sense r-erbAα primary transcript splicing into r-erbAα2 mRNA by RNA masking, thus tilting the balance towards R-erbAα1 synthesis and ultimately modulating cellular response to hormone [6,22,23,31,34]. The inhibition of splicing by NAT complementary to sequences remote from the splice site is specific and efficient, which might be attributed to blocking of regulatory elements within the exon essential for exon selection and intron removal, disruption of pre-mRNA secondary structure important for splicing, or disruption of RNP structure required for assembly of a functional RNA-splicing complex [22,35]. As a result, NAT dictates the way how a sense transcript is differentially spliced. A further analysis of the 414 NATs pairs involving splicing variants with different overlaps may help uncover underlying mechanisms.

Also in the above example, the antisense transcript encodes protein Rev-erbAα (NR1D1) which happens to belong to the thyroid/steroid hormone receptor family, same as products of sense transcripts [9]. This example illustrates the dual roles of some NATs: template for translation and regulator of sense gene expression. Such a gene structure is quite exquisite and economic to organize functionally related genes.

### One-to-many relationship in hNATs

**Figure 1:**
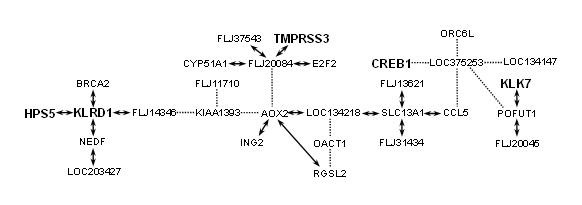
**Human NAT interaction network constituted by one-to-many relationship**. Gene symbol represents a gene. Boldface gene symbols represent genes with variants which could pair with a same countertranscript. A dashed line indicates an uncertain type due to lacking genomic mapping data; a solid line with bi-directional arrows indicates a *trans *type. There is no *cis*-NAT shown in this figure.

Initially, NAT pairs were considered to have one-to-one relationship. However, an antisense transcript might form duplex with more than one splice variants of a sense gene, as the examples given in previous section. In theory, an antisense transcript has also the potential to pair with more than one paralogous sense transcripts. Finally, more than one antisense transcripts may pair with one sense transcript at different parts of its sequence. In the 568 hNAT pairs, besides the 168 entries that form one-to-many relationship caused by alternative splicing, additional 97 hNAT pairs were found to have one-to-many relationship due to different pairing regions. That makes 47% ((97+168)/568) of the total, allowing NATs to form complex regulation networks. Figure 1 is an example of such a network including 29 genes involved in 40 hNATs. Interestingly, all hNAT pairs with genomic mapping data are *trans *type. In fact, hNAT pairs in this paper formed 38 such networks in various sizes (see Additional Table 3). It is worth noting that these networks mainly involve *trans *NATs, same as the example in Figure 1. The *trans *relationship seems more flexible, thus provides greater chance for transcripts to "communicate" with each other. In addition to RNA masking mentioned above, there is now strong evidence that the interaction of antisense partners can also affect gene expression via the activation of dsRNA-dependent pathways [22,36]. These might include RNA interference (RNAi)-dependent gene silencing. It was reported that several different micro RNAs could regulate the expression of the same target mRNA based on complementarity between micro RNA and mRNA [21]. It seems that the one-to-many relationship, notably in transcriptional regulation, might be common as the list of known one-to-many regulatory interactions becomes more comprehensive. From this perspective, the biological significance of *trans *NATs deserves to be further investigated systematically.

### Identification of two hNAT pairs with inverse expression pattern

There have been reports that sense and antisense transcripts show inverse expression pattern, as well as examples of coordinated regulation of both transcripts [9,10,12,37]. It is more intriguing that sense and antisense transcripts are differentially expressed depending on tissue types or development stages [25,38,39]. All these phenomena illustrated that the underlying mechanism is complicated and confusing. In this work we focused on identifying inverse expression pattern under certain condition for the hNAT pairs we found, considering that this pattern is the most common one for sense-antisense expression.

Microarray is a high-throughput technology for analyzing gene expression and has been recently applied to study NATs [12]. In this work, we used data from SMD [19] for *in silico *expression analysis of the hNATs we found. Rome *et al*. [40]designed a microarray of 29308 cDNA probes to evaluate gene expression pattern of skeletal muscle cells from six independent volunteers before and after insulin injection. We found that 150 out of the 568 NATs reported in this work had representing probes in the array, so we selected the expression data of 121 NATs with no less than 3 repeat samples for analysis. *T*-test and multiplicity adjustment showed that relative quantity (RQ) values of two NAT pairs, SARM1/MGC9564 (No. 550 in Additional Tables 1 and 4) and HARS/WDR55 (No. 443 in Additional Tables 1 and 4), varied significantly after insulin injection, showing the two pairs displayed inverse expression pattern. The calculation result is presented in Additional Table 4.

For the SARM1/MGC9564 pair, RQ value rose after insulin injection, indicating SARM1 is relatively up-regulated in contrast to MGC9564. SARM1 mRNA encodes a conserved protein with a SAM motif and is highly expressed in liver and kidney [41]. It was reported that a 0.4 kb antisense transcript was coordinately expressed with the SARM1 gene, but this is apparently not the same NAT as we found [41], suggesting that the SARM1 gene has at least two antisense partners. MGC9564 is an experimentally supported full-length mRNA (2096 bp) with a predicted coding sequence. According to the pairing data, SARM1 mRNA and MGC9564 mRNA possibly form a CDS vs. 3'UTR *cis *NAT pair.

For the second pair, HARS/WDR55, the relative quantity decreased after insulin injection, indicating HARS is down-regulated relative to WDR55. HARS mRNA codes for histidyl-tRNA synthetase, which is essential for the incorporation of histidine into proteins [42]. The WDR55 cDNA was cloned recently [43], and has not yet been subjected to final review in the latest release of NCBI RefSeq database. Based on the chromosomal location information, the two genes are mapped closely (5q31.3), but do not overlap. They may form a 3' UTR vs. 3' UTR *trans *NAT pair. This is a novel pair that we reported for the first time.

The apparent inverse expression pattern of the above two hNAT pairs suggests their possible regulatory roles in skeletal muscle cells after insulin injection. Further experimental studies are needed to unravel the underlying mechanism.

## Conclusion

Through a systematic analysis of hNATs using RefSeq dataset, we identified 568 hNATs. Even though the total number is less than those reported by Yelin *et al*. [12] and Chen *et al*. [13], many novel hNATs, particularly *trans *NATs, were discovered in this work. Among the NAT pairs involving splice variants, a remarkably high percentage (77.4%) have their pairing regions affected or completely eliminated by alternative splicing, suggesting significant relationship of alternative splicing and antisense-directed regulation. The extensive occurrence of splice variants in hNATs and other multiple pairing patterns results in one-to-many relationship, allowing the formation of complex regulation networks. It seemed that *trans *NATs bring more flexibility and complexity to antisense-directed gene regulation. Furthermore, out of the 121 hNATs with expression data available, we found that the expression pattern of two NAT pairs in the skeletal muscle cells showed significant inverse relationship before and after insulin injection.

In summary, NATs might carry out more extensive and complex functions than previously thought. Combined with endogenous micro RNAs, hNATs could be regarded as a special group of transcripts contributing to the complex regulation networks.

## Methods

### Data source

Human transcript sequences (25,827 in total) were extracted from RefSeq release 2 [18]. Genomic mapping data were taken from the Genome database version 35.1 at NCBI [44]. Alternative splicing information was obtained from AceView [33]. Microarray data used for *in silico *expression analysis were downloaded from Stanford Microarray Database (SMD) [19].

### Search for hNATs

The BLASTN program was used to identify putative hNATs with an e-value cutoff of 1e-9 and identity threshold of 98%. Query sequences were all human RNA sequences extracted from the RefSeq database, and the subject database was made up with reverse complement sequences of all query sequences. Option -S was set to be 1 to avoid the program reverse-complementing query sequences automatically. After getting BLASTN hits, we compared pairing sequence segments to the repetitive sequence database, RepBase [45], and confirmed that they contained no known repetitive sequence. We also used ClustalW to align all pairing segments to make sure there was no novel repeats. These two steps are necessary to eliminate the possibility of repetitive element-induced prevalence of hNATs.

### Classification of hNATs according to genomic location and pairing region

Human NATs were divided into *cis *and *trans *types according to relative genome location using genomic mapping data from the Genome database [44]. That is, the NAT transcribed from the opposite strand of the same genomic locus as its sense RNA is *cis*, while the NAT transcribed from a genomic locus different from its sense counterpart is *trans*. Based on the pairing region of sense and antisense transcripts, we also defined 6 types: 1) 5'UTR vs. 5'UTR, 2) 5'UTR vs. CDS, 3) 5'UTR vs. 3'UTR, 4) CDS vs. CDS, 5) CDS vs. 3'UTR, and 6) 3'UTR vs. 3'UTR, where the "CDS" contains "CDS", "CDS+3'UTR", "CDS+5' UTR" and "5'UTR+CDS+3' UTR".

### Expression analysis of hNATs using SMD data

Gene expression data of human skeletal muscle cells before and after insulin injection are available in SMD [19,40]. Since human cDNA probes in these data are identified with UniGene cluster IDs, antisense transcripts can be mapped to corresponding probes based on the relationship between UniGene cluster ID and RefSeq accession number (ACC), by which expression data of hNATs were retrieved. Human NATs with data from 3 or more repeat samples were selected for statistical analysis. The ratio of sense to antisense transcript expression levels represents the relative quantity (RQ) of a NAT pair, RQ = S/A. The change of the ratio of a pair after insulin injection is expressed as the ratio of two relative quantities, R = RQ_a_/RQ_b_, where RQ_b _and RQ_a _are relative quantities before and after insulin injection, respectively. This R value is used to check if a NAT pair shows an inverse expression pattern under the given condition. *T*-test with a significance level of 0.05 and Bonferroni's adjustment for multiplicity were used to evaluate the significance of an R value, that is, the change of a NAT pair in expression pattern.

## Authors' contributions

YY Li and L Qin designed the research pipeline, performed the statistical analysis and drafted the manuscript. ZM Guo, H Xu and J Su carried out the programming. L Liu, P Hao and YX Shi participated in the design of the study. WZ He and L Liu helped to draft the manuscript. YX Li conceived and oversaw the research. All the authors read and approved the final manuscript.

## Supplementary Material

Additional File 1 Table1_All hNATs identified in this study. Click here for file

Additional File 2 Table2A_hNATs with splice variants of genes. Click here for file

Additional File 3 Table2B_hNATs with splice variants of genes. Click here for file

Additional File 4 Table3_hNATs with potential to form networks. Click here for file

Additional File 5 Table4_Statistic analysis of microarray data of skeletal muscle. Click here for file

## Figures and Tables

**Table 1:** Summary of hNAT pairs

	***Cis***	***Trans***	**Lacking genomic data**	**Total**
**Novel**	156	157	90	403
**Reported**	156	4	5	165
**Total**	312	161	95	568

**Table 2:** Classification of hNAT pairs based on pairing region

	***Cis***	***Trans***	**Lacking genomic data**	**Total**
(1) 5'UTR vs. 5'UTR	20	7	5	32
(2) 5'UTR vs. CDS	37	16	13	66
(3) 5'UTR vs. 3'UTR	4	26	6	36
(4) CDS vs. CDS	48	1	22	71
(5) CDS vs. 3'TUR	85	19	23	127
(6) 3'UTR vs. 3'UTR	116	78	24	218
Lacking CDS	2	14	2	18
**Total**	312	161	95	568
